# The 2021 Western Germany flood event: The value of flood risk dissemination strategies and social media

**DOI:** 10.4102/jamba.v14i1.1322

**Published:** 2022-12-09

**Authors:** Newton Matandirotya

**Affiliations:** 1 Centre for Climate Change Adaptation and Resilience, Kgotso Development Trust, Beitbridge, Zimbabwe; 2 Department of Geosciences, Faculty of Science, Nelson Mandela University, Port Elizabeth, South Africa

## Introduction

On 12–15 July 2021, Western Germany was hit by devastating floods that resulted in the deaths of an estimated 180 people (Fekete & Sandholz 2021; Kreienkamp et al. 2021). Germany does have a history of flood events, with the most recent and prominent being in 2013 over the regions of Elbe and Danube (Kuhlicke et al. 2020), leading to an estimated loss of EUR 6.8 billion (Kuhlicke et al. 2020). Moreover, another notable flood event is the June 2016 event over Bavaria (Mayr, Thaler & Hübl 2020). Globally floods remain a leading natural hazard that many communities remain vulnerable to (De Boer 2014) as the frequency and intensity is also increasing because of climate change and urbanisation, especially for those residing within delta basins, river banks and coastal regions (Haer, Bozen & Aerts 2016; Jongman, Ward & Aerts 2012). It is estimated that between 2000 and 2019, floods have resulted in 100 000 deaths, caused an estimated USD 651bn in economic losses and negatively impacted 1.6 billion people worldwide (Koç, Natho & Thieken 2021).

During the July 2021 flood event, the European Flood Alert System did some modelling on the 09-12 July 2021 while the German Weather Broadcasting Agency issued weather warning with some messages being posted on social media platforms (Fekete & Sandholz 2021). Accurately predicting flood events remains a challenge, as these are rare in nature; for example, they have a probability of approximately 8% of occurring three times in a period spanning 100 years (Cloke & Pappenberger 2009), and hence, sometimes prediction uncertainties become inevitable and can result in system failures. It is the purpose of flood forecasting to avail reliable and accurate information that enables alerts and warnings to be issued and communicated (Kundzewicz 2017) well in advance. Flood forecasting depends fundamentally on the hydrological and meteorological laws of floods (Zhou et al. 2021). A well functioning flood early warning system can reduce the levels of economic losses (Budimir et al. 2020); however, several barriers still exist that relate to accessing early warning information, processing it, understanding it and taking some action that allows communities to prevent the catastrophic effects of flood events (Budimir et al. 2020). In addition, challenges remain on how warning messages are communicated to communities (Grothmann & Reusswig 2006; Intrieri et al. 2020) to make them useful for the reduction of flood-related disasters (Budimir et al. 2020) through early warning systems (EWS). Early warning systems are regarded as the capacity to generate and disseminate potential hazard information that facilitates mitigation against harm by individuals, households and communities (Cools, Innocenti & O’Brien 2016).

The main objective of EWS is to reduce human life and economic losses (Kreibich, Hudson & Merz 2021; Potter, Harrison & Kreft 2021) through the provision of information that allows action by individuals and communities before the emerging hazard has struck (Rana et al. 2021). Early warning systems are usually composed of four pillars, namely risk knowledge, monitoring, dissemination and communicating (Budimir et al. 2020; Cools et al. 2016). The majority of Flood Early Warning Systems (FEWS) are anchored on observational precipitation data, which is normally gathered through surface-based measurements, for example, radars and rain gauges, which are activated when certain thresholds are reached (Cloke & Pappenberger 2009), while on the other hand, numerical weather models can be used to make a future forecast of 2–15 days ahead (Cloke & Pappenberger 2009). The ability to properly deal with and manage disasters revolves around how communities are organised, as well as how knowledge is interpreted, communicated and used (Albris, Cedervall & Emmanuel 2020; Cools et al. 2016). It is also inevitable that wrong scientific information can lead to dire consequences that result in loss of life or economic losses (Albris et al. 2020). Further challenges can also emerge from how flood risk information communicated is utilised at the individual, household and community levels; for example, in the United Kingdom, a study by Amaratunga et al. (2017) established that even though equipped with appropriate awareness, sometimes communities choose their own paths contrary to scientific advice. Germany is a member of the European Flood Awareness System (EFAS) mandated to do preparatory work and offer warnings before the outbreak of major flood events (Demeritt et al. 2013), although the July 2021 event seems to have put the EFAS system capability to the test.

In addition, other obstacles are also a result of finding the right balance in how to best do risk awareness before a hazard event, as poor communication has been cited as one of the major impediments (Rana et al. 2021). Furthermore, with the rise of other means of communication such as social media, challenges emerge in how messages are packaged and how well they are communicated to communities, while also battling the phenomenon of fake news (Reuter, Hughes & Kaufhold 2018). The integration of social media and mainstream media can be harnessed to enable effective flood risk awareness. This article’s commentary puts into perspective the challenges in communicating early warning messaging. This commentary used secondary data based on literature review, while meteorological data were sourced from the Wetterzentrale website.^1^ The commentary is composed of five sections. Section 2 describes the weather conditions between 12 and 15 July 2021; Section 3 outlines the importance of applying the people-centred approach; Section 4 showcases and outlines alternative flood warning communication tools; and Section 5 concludes with future FEWS policymaking prospects.

## Meteorological conditions during days of floods

**FIGURE 1:**
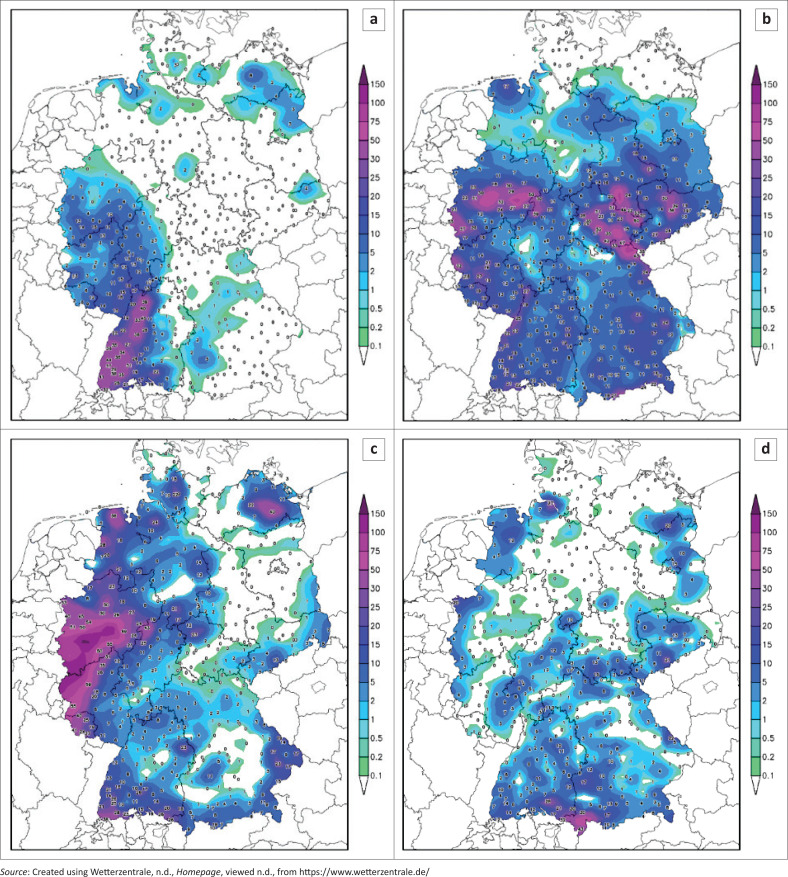
Total 24-h precipitation received across Germany: (a) 12 July 2021, (b) 13 July 2021, (c) 14 July 2021, (d) 15 July 2021.

Figure 1 highlights the total 24-h precipitation for the days from 12 July 2021 to 15 July 2021. The most affected region was Western Germany, which received the highest levels of precipitation over the 4 days. The highest precipitation was received on 14 July 2021, as highlighted in Table 1. The highest amount of rainfall was received at the Rheinbach-Todenfeld weather station, totalling 158 mm (Table 1).

**TABLE 1:** Weather stations that received the highest precipitation from 14 to 15 July 2021.

Station	Precipitation received (mm)
Rheinbach-Todenfeld	158
Cologne-Stammheim	154
Klein-Altendorf	147
Kall-Sistig	145

*Source:* Deutsche Welle (DW), 2021, *Germany mulls manslaughter probe into deadly floods*, viewed from https://www.dw.com/en/germany-mulls-manslaughter-probe-into-deadly-floods/a-58734289

Table 1, to be read with Figure 1, provides details of the maximum amount of precipitation received during the period 14–15 July 2021. The station that received the highest amount of precipitation was Rheinbach-Todenfeld, although all the regions that received high precipitation were all located in the Western region (Figure 1).

**FIGURE 2:**
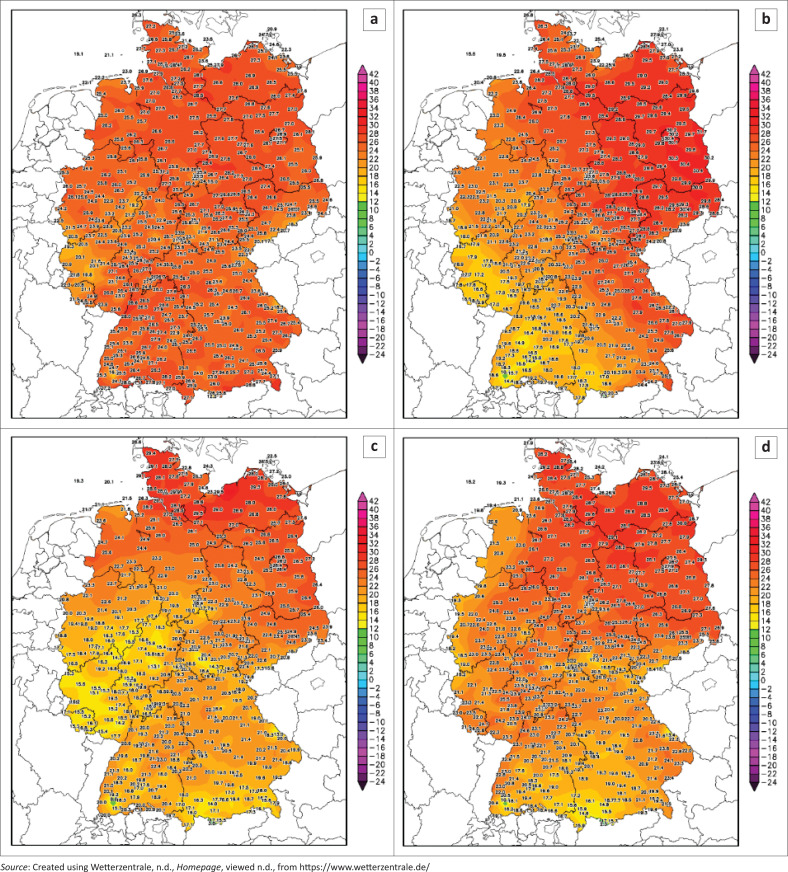
Maximum temperature across Germany during the days of floods: (a) 12 July 2021, (b) 13 July 2021, (c) 14 July 2021 and (d) 15 July 2021.

Figure 2 shows the maximum temperature across Germany, showing that the highest temperatures were recorded on 12 and 13 July 2021. The high surface temperature was coupled with very low pressure that created ideal conditions for rainfall formation.

## Application of people-centred flood risk communication and information dissemination strategies

According to Cools et al. (2016), for an early warning system to be effective, there is a need to set up engagement platforms that bring together government authorities and local community members where information and plans can jointly be shared. Furthermore, there is also a need to adapt communication strategies to local conditions that facilitate for communities to act quickly on forecasted information; otherwise, if there is no action, communities are likely to suffer losses (Cools et al. 2016). This can be achieved through the adoption of people-centred participatory approaches (Potter et al. 2021) that capacitate individuals and communities to have sufficient time to act and avoid hazard damage. On the other hand, Demeritt and Nobert (2014) advocate the simplification of language used when crafting messages and avoiding the use of jargon and even strong statistics, therefore allowing that there are misinterpretations and misunderstandings. Early local community stakeholder engagement can also remove potential misunderstandings that can arise when only ‘experts’ are involved, which can potentially lead to resentment (Intrieri et al. 2020). Furthermore, Demeritt and Nobert (2014) also highlight the importance of trust in the messenger, as this will facilitate the utilisation and action by communities unlike in circumstances where there is a lack of trust. Traditionally, risk communication has been done through government agencies that rely on certain bureaucratic protocols and are top-down in nature (Haer et al. 2016; Mustafa et al. 2015). In several jurisdictions, this is not very effective, as it lacks end-user participation in the chain (Mustafa et al. 2015). It might thus be time to open up the space to other development actors and remove the veil of what can be communicated, how it is communicated and when it is communicated. Through liberalising the space, community engagement can thus be increased and suspicion reduced.

The involvement and engagement of people early on allow communities opportunities to make self-assessments and informed decisions based on their peculiar circumstances. Also, Haer et al. (2016) advocate the use of a model that makes use of social networks to influence overall community behaviours, for example, social peer pressure. Furthermore, Intrieri et al. (2020) advocate for alert messages that are packaged such that they contain additional explanatory information instead of relying on colour codes only, and the same view is also expressed in Potter et al. (2021), who encourages the avoidance of generic and static threshold warnings that do not move with the times. The same authors also encourage that risk communication should come with infographics, especially when disseminated through social platforms. These technologies can be exploited as ways to reduce flood negative impacts. Moreover, all alert levels should be tailored to certain channels of communication that are also reflective of the level of danger, for example, resorting to megaphones, sirens and loudspeakers with the participation of local community leaders to highlight the level of emergency for evacuation purposes. Also, measures should be put in place that curb false warnings and in the long run also prevent warning fatigue (Potter et al. 2021). Furthermore, Villagrán de León et al. (2006) propose a people-centred FEWS that incorporates four arms, namely risk knowledge, warning service, dissemination and response capability.

## Complementary flood mitigation tools: Social media and artificial intelligence

Social media remains a very powerful, useful communication platform during times of disasters, particularly looking at the number of users; for example, as of 2021, Facebook had 2.89 billion users, 2.3 billion for YouTube, 2 billion for WhatsApp and 1.4 billion for Instagram,^2^ thus making it a very valuable resource in times of disasters. This can thus become a valuable resource that can be tapped into for purposes of flood risk communication (Intrieri et al. 2020). In Germany, however, a study by Reuter et al. (2017) indicated that 79% of respondents had not downloaded a crisis warning application before, while 16% confirmed downloading and using such an application, including 4% who use the Notfall-Informations und Nachrichten-App (NINA). There is also a need for finding ways to bridge the gap between technical warning language and messaging that affords communities to understand and quickly act on warning messages, and this way loss of human life and economic losses can be reduced. Furthermore, with the advent of social media, authorities need to also utilise the power embedded in such networks to improve risk management while also putting some safeguards in place to avoid the spread of false information. Mitigation can also be enhanced through the integration of artificial intelligence (AI), remote sensing and social media (Perera et al. 2020), for example, the use of AI-related tools such as artificial neural networks (ANNs) and fuzzy inference systems (FISs) (Nur Adli Zakaria et al. 2021). These tools have extensively been used to monitor river levels, therefore presenting an opportunity for authorities to tap into their power as mitigation tools in reducing flood negative impacts.

## Future prospects for flood early warning systems policymaking

The July 2021 Germany flood event highlighted the need for the world to be ready for the regular occurrence of extreme flood events in the future because of climate change (Leal Filho et al. 2021). Flood EWSs should be matched with the empowerment of communities to adequately respond timeously to warnings (Villagrán de León et al. 2006). Furthermore, FEWS activities can further be strengthened through adopting multisectoral communication and multipronged pathways for effective early flood risk messaging. Moreover, flood forecasters, disaster risk reduction (DRR) practitioners and policymakers can also utilise other complementary tools such as AI and remote sensing to enhance future predictions. Moreover, DRR authorities can also tap into the large audience reach that lies within social media platforms to enhance citizens’ flood awareness levels. There is always, however, need to guard against flood misinformation and the spread of fake news, particularly over social media platforms. In Germany, the low uptake of social media applications can be an opportunity to promote these as alternative sources of early warnings. It is imperative that going forward, flood events should be tackled using a suite of tools to save lives and reduce economic damage.

## Footnotes

1 See https://www.wetterzentrale.de/reanalysis.

2 See https://www.statista.com/statistics/272014/global-social-networks-ranked-by-number-of-users/

## References

[CIT0001] Amaratunga, D., Malalgoda, C., Haigh, R., Panda, A. & Rahayu, H., 2018, ‘Sound Practices of Disaster Risk Reduction at local level’, *Procedia Engineering* 212, 1163–1170. 10.1016/j.proeng.2018.01.150

[CIT0002] Albris, K., Cedervall, K. & Emmanuel, L., 2020, ‘Disaster knowledge gaps: Exploring the interface between science and policy for disaster risk reduction in Europe’, *International Journal of Disaster Risk Science* 11(1), 1–12. 10.1007/s13753-020-00250-5

[CIT0003] Budimir, M., Donovan, A., Brown, S., Shakya, P., Gautam, D. & Uprety, M. et al., 2020, ‘Communicating complex forecasts: An analysis of the approach in Nepal’s flood early warning system’, *Geoscience Communication* 3(1), 49–70. 10.5194/gc-3-49-2020

[CIT0004] Cloke, H.L. & Pappenberger, F., 2009, ‘Ensemble flood forecasting: A review’, *Journal of Hydrology* 375(3–4), 613–626. 10.1016/j.jhydrol.2009.06.005

[CIT0005] Cools, J., Innocenti, D. & O’Brien, S., 2016, ‘Lessons from flood early warning systems’, *Environmental Science and Policy* 58, 117–122. 10.1016/j.envsci.2016.01.006

[CIT0006] De Boer, J., Wouter Botzen, W.J. & Terpstra, T., 2014, ‘Improving flood risk communication by focusing on prevention-focused motivation’, *Risk Analysis* 34(2), 309–322. 10.1111/risa.12091 23834075

[CIT0007] Demeritt, D. & Nobert, S., 2014, ‘Models of best practice in flood risk communication and management’, *Environmental Hazards* 13(4), 313–328. 10.1080/17477891.2014.924897

[CIT0008] Demeritt, D. Nobert, S., Cloke, H.L. & Pappenberger, F., 2013, ‘The European flood alert system and the communication, perception, and use of ensemble predictions for operational flood risk management’, *Hydrological Processes* 27(1), 147–157. 10.1002/hyp.9419

[CIT0009] Deutsche Welle (DW), 2021, *Germany mulls manslaughter probe into deadly floods*, viewed 02 February 2022, from https://www.dw.com/en/germany-mulls-manslaughter-probe-into-deadly-floods/a-58734289.

[CIT0010] Fekete, A. & Sandholz, S., 2021, ‘Here comes the flood, but not failure? Lessons to learn after the heavy rain and pluvial floods in germany 2021’, *Water (Switzerland)* 13(21), 1–20. 10.3390/w13213016

[CIT0011] Grothmann, T. & Reusswig, F., 2006, ‘People at risk of flooding: Why some residents take precautionary action while others do not’, *Natural Hazards* 38(1–2), 101–120. 10.1007/s11069-005-8604-6

[CIT0012] Haer, T., Botzen, W.J.W. & Aerts, J.C.J.H., 2016, ‘The effectiveness of flood risk communication strategies and the influence of social networks – Insights from an agent-based model’, *Environmental Science and Policy* 60, 44–52. 10.1016/j.envsci.2016.03.006

[CIT0013] Intrieri, E., Dotta, G., Fontanelli, K., Bianchini, C., Bardi, F. & Campatelli, F. et al., 2020, ‘Operational framework for flood risk communication’, *International Journal of Disaster Risk Reduction* 46, 101510. 10.1016/j.ijdrr.2020.101510

[CIT0014] Jongman, B., Ward, P.J. & Aerts, J.C.J.H., 2012, ‘Global exposure to river and coastal flooding: Long term trends and changes’, *Global Environmental Change* 22(4), 823–835. 10.1016/j.gloenvcha.2012.07.004

[CIT0015] Koç, G., Natho, S. & Thieken, A.H., 2021, ‘Estimating direct economic impacts of severe flood events in Turkey (2015–2020)’, *International Journal of Disaster Risk Reduction* 58, 102222. 10.1016/j.ijdrr.2021.102222

[CIT0016] Kreibich, H., Hudson, P. & Merz, B., 2021, ‘Knowing what to do substantially improves the effectiveness of flood early warning’, *Bulletin of the American Meteorological Society* 102(7), E1450–E1463. 10.1175/BAMS-D-20-0262.1

[CIT0017] Kreienkamp, F., Philip, S.Y., Tradowsky, J.S., Kew, S.F., Lorenz, P. Arrighi, J. et al., 2021, ‘Rapid Attribution of Heavy Rainfall Events Leading to the Severe Flooding in Western Europe during July 2021’, *World Weather Atribution*, viewed n.d., from http://hdl.handle.net/1854/LU-8732135.

[CIT0018] Kuhlicke, C., Masson, T., Kienzler, S., Sieg, T., Thieken, A.H. & Kreibich, H., 2020, ‘Multiple flood experiences and social resilience: Findings from three surveys on households and companies exposed to the 2013 flood in germany’, *Weather, Climate, and Society* 12(1), 63–88. 10.1175/WCAS-D-18-0069.1

[CIT0019] Kundzewicz, Z.W., 2013, ‘15 Floods: lessons about early warning systems’, in European Environment Agency (EEA) (ed.), *Late lessons from early warning: science, precaution, innovation*, pp. 347–368, EEA report no 1/2013, European Environment Agency, Copenhagen.

[CIT0020] Kundzewicz, Z.W., 2017, ‘15 floods : Lessons about early warning systems’, *Emerging Lessons from Ecosystems* 347–368. 10.1007/978-1-4020-4399-4

[CIT0021] Leal Filho, W. Matandirotya, N.R., Lütz, J.M., Abate Alemu, E., Brearley, F.Q., Ago Baidoo, A. et al., 2021, ‘Impacts of climate change to African indigenous communities and examples of adaptation responses’, *Nature Communications* 12(1), 13–16. 10.1038/s41467-021-26540-0 PMC8553734 34711857

[CIT0022] Mayr, B., Thaler, T. & Hübl, J., 2020, ‘Successful small-scale household relocation after a millennial flood event in Simbach, Germany 2016’, *Water (Switzerland)* 12(1), 156. 10.3390/w12010156

[CIT0023] Mustafa, D., Gioli, G., Qazi, S., Waraich, R., Rehman, A. & Zahoor, R., 2015, ‘Gendering flood early warning systems: The case of Pakistan’, *Environmental Hazards* 14(4), 312–328. 10.1080/17477891.2015.1075859

[CIT0024] Nur Adli Zakaria, M., Abdul Malek, M., Zolkepli, M. & Ahmed, A., 2021, ‘Application of artificial intelligence algorithms for hourly river level forecast: A case study of Muda River, Malaysia’, *Alexandria Engineering Journal* 60(4), 4015–4028. 10.1016/j.aej.2021.02.046

[CIT0025] Perera, D., Seidou, O., Agnihotri, J., Mehmood, H. & Rasmy, M., 2020, ‘Challenges and technical advances in flood early warning systems (FEWSs)’, in G. Huang (ed.), *Flood impact mitigation and resilience enhancement*, pp. 1–18, IntechOpen, London. 10.5772/intechopen.93069

[CIT0026] Potter, S., Harrison, S. & Kreft, P., 2021, ‘The benefits and challenges of implementing impact-based severe weather warning systems: Perspectives of weather, flood, and emergency management personnel’, *Weather, Climate, and Society* 13(2), 303–314. 10.1175/WCAS-D-20-0110.1

[CIT0027] Rana, I.A., Bhatti, S.S. & Jamshed, A., 2021, ‘Effectiveness of flood early warning system from the perspective of experts and three affected communities in urban areas of Pakistan’, *Environmental Hazards* 20(3), 209–228. 10.1080/17477891.2020.1751031

[CIT0028] Reuter, C., Hughes, A.L. & Kaufhold, M.A., 2018, ‘Social media in crisis management: An evaluation and analysis of crisis informatics research’, *International Journal of Human-Computer Interaction* 34(4), 280–294. 10.1080/10447318.2018.1427832

[CIT0029] Reuter, C., Kaufhold, M., Leopold, I. & Knipp, H., 2017, ‘Katwarn, NINA or FEMA? Multi-method study on distribution, use amd public views on crisis apps’, in Proceedings of the 25th European Conference on Information Systems, ECIS 2017, Guimaraes, Portugal, June 5–10, 2017, pp. 2187–2201.

[CIT0030] Villagrán de León, J.C., Bogardi, J., Dannenmann, S. & Basher, R., 2006, ‘Early warning systems in the context of disaster risk management’, *Entwicklung and Ländlicher Raum* 2, 23–25, viewed n.d., from http://www.unisdr.org/eng/sasakawa/.

[CIT0031] Wetterzentrale, n.d., *Homepage*, viewed n.d., from https://www.wetterzentrale.de/

[CIT0032] Zhou, F., Chen, Y., Wang, L., Wu, S. & Shao, G., 2021, ‘Flood forecasting scheme of Nanshui reservoir based on Liuxihe model’, *Tropical Cyclone Research and Review* 10(2), 106–115. 10.1016/j.tcrr.2021.06.002

